# Risk analyses in the intersection between patient and workplace safety: A case study of hazids in para-clinical supporting systems in specialized health care

**DOI:** 10.1177/2050312120977136

**Published:** 2020-11-27

**Authors:** Marianne Palm, Leif Moen, Geir Sverre Braut

**Affiliations:** 1 Oslo University Hospital, Oslo, Norway; 2 Stavanger University Hospital, Stavanger, Norway

**Keywords:** Risk analysis, hazid, support systems in hospitals

## Abstract

**Background:**

The aim is to investigate the appropriateness of hazid for performing risk analyses in supporting systems in hospitals.

**Methods:**

We used a case study approach for evaluating introduction of hazid for the first time in two different university hospital settings. The hazid was performed in a customized way according to the specific needs at the two sites.

**Findings:**

In both settings studied, the hazid approach revealed several phenomena that were followed up in the ordinary quality improvement work. The results were widely acknowledged as valid as seen from the managerial level. The participants reported that they felt comfortable in the hazid process and were able to freely present their current concerns and perspectives on risks related to their daily work.

**Conclusion:**

Hazid is basically a meeting between competent workers who elaborate on their own risk picture. It is giving other types of information not gained through other often-used approaches. Specific risk factors can be described in real time as seen by people directly involved, thus circumventing the hierarchy in the organization. The process in itself can trigger improvement actions.

**Application to practice:**

Hazid can be used for presenting a valid risk picture as seen from below in the organization.

## Introduction

In recent decades, providers of health care have increasingly been challenged to present risk pictures as a basis for decisions related to patient, as well as workplace, safety. To some extent, this is based on legal requirements.^1,2^ Different approaches for performing risk analyses to be used at a higher organizational, as well as at an operational, level have been suggested and, to some degree, even scientifically tested.^3^

In provision of health care, there has been a strong focus on risk assessments based on empirical data, for example, information from unexpected and unwanted incidents.^4^ Typically, data to be used in prospective risk analyses build upon experiences from a root-cause analysis of minor or major incidents.^5^ The predominant model for risk analyses seems to be some variation of preliminary hazard analysis, ending with the well-known risk matrix. This approach has also become popular and widely used for prospective risk analyses in systems providing public services outside the health sector, and for general societal planning purposes.^6^ In the toolkit for risk analysts, there are several other methods available. Many of them are scarcely used and poorly tested in health care settings. Thus, there are alternatives to the root-cause analyses and the risk matrices, but they often have to be constructed for each new system.^7^

Hazid is one of the methods available.^8^ It enables experiences from personnel at the practical level to be actively used in continuous service development.

The aim of this study was to explore if customized versions of the hazid method can be appropriate tools for performing risk analyses at an operational level in para-clinical supporting systems in hospitals. In this project, we tested the feasibility of using it in customized ways in two different hospital settings.

### Theoretical foundation

Hazid is a well-known technique for identifying and assessing risks in industry and communication systems.^8^ Although performed with some variations in different settings, it can be regarded as a slightly simplified variant of the somewhat more structured hazop method.^9,10^ It is also closely related to preliminary hazard analysis, although not focusing on probability or likelihood as much as is usual in this type of analysis.

Risk is often defined as the combination of the probability of an event and its consequences. By this approach, rarely occurring events tend to be omitted from further analysis due to the low compound risk associated with them. The low risk will tend to pull them away from the “red area” in the risk matrix, which also often takes them away from the attention of managers and professionals in their systematic work for enhancing safety. Still, we know that incidents with a low probability may cause severe accidents.^11^

Currently, there is an ongoing debate on the concept of uncertainty in medicine and health care.^12–15^ Probability is only one way of approaching uncertainty. From statistics, we learn that probability is the ratio of the number of outcomes of a certain kind to the total number of possible outcomes, where the number of possible outcomes is infinite. Often we are not in the position, neither in practice nor in theory, to test or model such situations. We have to deal with single patients, and we have to approach unique systems. Then, we are not able to present data for making valid and reliable forecasts on what will happen in the near or far future. The possibility to attribute relevant probabilities to the consequences is restricted.

By means of hazid, the concept of uncertainty can be approached in a qualitative way, not leading to more-or-less futile discussions on probabilities based on poor data available.^8^ Hazid is commonly performed at ground level in an organization, as it aims to extract practical, risk-related information from those persons working directly on the practical issues.^16^

Incident reporting systems—which are widely used in health care, for example, in root-cause analyses—are focusing on particular deviations, accidents and so on trying to elaborate on the causes behind the incident reported. The hazid approach is also occupied with those issues, but it opens up the situations for thoughts and discussions “the other way around.” It encourages the participants to present their experiences, doubts, beliefs and uncertainties connected with their own day-to-day challenges at the workplace, thus elaborating on consequences of different types of activities. From this background, one may try to establish what is to be regarded as normal (good) practice, and at which points we have to be aware of possible deviations connected with increasing risk of incidents or damages.

### Para-clinical supporting systems

In this study, we have chosen two different para-clinical supporting systems. Such systems we define as those related to the “supply chain” of the hospital—with direct impact on patient safety, but without taking part in clinical judgments and decisions related to the individual patient.

We have chosen para-clinical supporting systems for this study for three reasons. Such systems often show the linear characteristics found in systems designed for performing work in industry and transportation. That is, by defining more or less limited algorithms and working processes, little room is left for professional considerations and decisions based on discretion made by single employees. In this way, these services are different from the clinical core services, performed by, for example, nurses and doctors, where the need for case-specific judgments is usually more prominent.

These supporting systems also often seem to be governed by normative procedures imposed from above, without coupling them to relevant feedback loops where practical experience from below is used for evaluation and adjustment of the practices. In this way, they are typically at the bottom of the organizational hierarchy of the hospital.

The third reason for choosing these two systems is that they deal with intersections in the causal chain of safety in the hospital. On the basis of their daily work, they may have knowledge and make observations important for the risk picture in the hospital, outside their own core area of responsibility. As they do not oversee the whole supply chain, they may not recognize the importance of their own experiences. This is a phenomenon worth studying in itself, cfr. experiences of accidents in other sectors.^11^

Both hospitals have extensive systems for quality management and patient safety protection. In both systems studied, regular risk analyses have been performed according to the risk matrix model, for several years, under the responsibility of the management.

## Methods

### Case study design

We have chosen a case study design. Our aim was to evaluate the feasibility of hazid in hospital settings and to gain experience on what kind of adjustments are needed for customizing the method for different settings in health care.^17,18^ The written reports from the hazids were examined using content analysis.

Our two chosen systems for this study were the hospital porter service (part A) and the supply chain of sterile equipment for surgical treatment (part B). The two cases were from two different Norwegian university hospitals. In both the systems studied, a majority of the employees had received some kind of formal education specific to their work, typically at the high-school level.

As the aim of this study is to report experiences with the use of the hazid method, the specific results gained from the two cases do not appear to be of general interest, as they due to contextual factors have low external validity. Therefore, they will not be presented here.

### Ethical and legal considerations

Both part A and part B were carried out as regular quality development activities governed by the line management in the respective systems. The participation in the hazid groups was voluntary and the participants were informed that the aims of the activity were twofold: achieving input information in the regular quality development work and testing a new method for engaging employees in risk analyses.

No electronic devices were used for collecting data in the hazid groups. Neither was any information related to the participating individuals stored or noted. Data on patients were not used. Therefore, the project could legally be performed on the basis of a managerial decision, without obtaining any external ethical or legal permissions.

### Performing the hazid

The hazid process was led by a consultant (G.S.B.), who was not part of the systems studied. In both parts, the leader of the process gave a short introduction on the method, emphasizing that it was the experiences from the participants in the groups that should be regarded as knowledge of interest. The questions and topics posed by the leader were intended to be as neutral as possible, merely stimulating the narrative potential of the group members. This is in line with the use of guidewords in the traditional hazop analyses.^8^

Examples of such guiding questions were as follows:

- Could you describe what happens from x to y (in the working process)?
- According to your judgment, where are the vulnerable moments (in this process)?
- Explain why you regard (this) as a risky situation?
- What can (phenomenon) lead to?

Part A was held about 1 month before part B to provide the opportunity to make adjustments according to experience gained in part A, which methodologically we regarded as a “piloting” activity. The only changes made between parts A and B were that in part B, the same group met once again to exchange experiences from the first meeting, and in part B, we also collected written feedback from the participants. The results from part A were plotted and analyzed by G.S.B. and L.M. M.P. and G.S.B. took care of the data from part B. All invited participants accepted to take part in the study.

Part A relating to the hospital porter system focused on conditions perceived by the porters as threats to patient safety and thus imposing increased practical and mental workload for the employees. Two different hazid groups were arranged, with three and four active participants, respectively, all of them working closely together. In addition, the elected representative for the porters was present, as well as one of the superiors. The task set for these two hazid groups was to identify possible factors observed by the porters that could influence patient safety in a negative way.

In part B, relating to the supply chain of sterile equipment for surgical treatment, a similar group meeting was arranged, this time with six participants. The participants were working in the same department, but on four different sites. The main task for this hazid process was to identify risk points in the supply chain for ensuring quality of services. In this group, few employees were working directly together. In part B, the head of the department made the subsequent report.

In both parts, the draft reports were presented to the participants in the hazid process by L.M. (part A) and M.P. (part B) before finalizing them, inviting the participants to clarify possible mistakes or suggest adjustments. Only minor adjustments were suggested in part A, and none in part B.

After taking part in the two meetings in part B, every participant also got a paper-based questionnaire, which they were asked to fill in anonymously and returned to M.P. on paper, without possibility of tracing the individual source. The time frame for the meetings was one-and-a-half hours. In both parts A and B, the discussion in the meetings seemed to reach saturation after just over 1 h. The group discussions were then closed after agreement between the leader and the participants.

## Results

The approved reports were examined using content analysis. The results from these analyses were used in the ordinary safety and quality assurance efforts in each of the involved departments. The content seems to have been accepted as valid and relevant by the management as well as the employees.

As the aim of this article is to present and discuss the method used, we shall not go into details on the findings related to specific aspects of the two para-clinical supporting systems studied. In the reports from both parts, there are several specific elements described that, according to the participants, could interfere with patient, as well as workplace, safety. Many of the factors that emerged from the hazid process had not previously been explicitly addressed in risk analyses or established procedures.

In the rest of this article, we will present and discuss some findings characterizing the hazid method and show its potential when used in ordinary safety improvement efforts in hospitals. Examples of findings of general interest are challenges related to communication of clinical information to the porters so that they could be prepared to take actions immediately in emergency situations (part A), and the lack of effective procedures concerning washing and control of instruments to be provided to the surgical departments (part B).

Common traits of the vulnerable situations described in both parts of the study are related to transferal of information at intersections, for example, through hand-over procedures. Both systems studied are characterized by a high degree of interdependence with clinical departments in the hospital.

In both settings, the participants commented that they had to acknowledge they were not a part of what is commonly regarded as at the core of the system providing health care in hospitals. Still, they were highly aware of being elements of this system, and that their actions impacted on patients’ safety. In both parts, it was commented that the participants valued the opportunity to give their opinions on judgments of safety related to the systems in which they worked, not least the opportunity to present their experiences in a minor group without the daily, normal managerial expectations from above.

In the written feedback from the participants in part B, four of six persons felt they had received sufficient information before the first group interview started. All of them, though, claimed they were satisfied with information on the method given in the interview. Except for one participant, everyone thought the group size is appropriate. All participants commented positively on the questions on perceiving the issues that emerged through the process relevant to their jobs, that it was “safe” to present their own opinions in the group, and that their opinions were taken seriously. Two of the six, though, felt the interview was too heavily led by the external consultant.

Overall, the participants reported that they were comfortable with their participation in the process and that the issues that emerged were relevant for their work. They also found it interesting to share their own experiences as well as getting feedback on their own working situation.

## Discussion

Both the studied para-clinical supporting systems are elements of a greater, knowledge-based health care system. Compared to the average employee in the clinical departments, the formal competence of the employees in these supporting systems is low. Still, their work tasks are critical elements in providing safe service systems for patients as well as employees.

The content of the work of the employees in these systems seems to not be well known by their colleagues in the clinical departments. Therefore, it may be a challenge for other professionals, the managers included, to perform risk analyses related to their work. This is a very relevant argument for training employees in these supporting systems to be aware of risks related to their tasks, for them to be able to make observations and judgments related to safety, and for them to have the boldness to describe and present them to their superiors and colleagues in other departments.

The participants were all ground-level employees without managerial tasks. Still, they showed a high competence in understanding how the wider systems in the hospital functioned. This should not come as a surprise, as the studied para-clinical support systems interrelate with a multitude of the clinical subsystems in the hospital.

However, the experiences of these employees have scarcely been used as sources of information related to safety and risk in the hospital. Perhaps, this could be the result of a strong hierarchy inside the hospital, where employees in these groups tend to be at a low level in the organization, and where their opinions are not demanded and their own boldness for presenting them is low. This may culminate in risk-related experiences going unnoticed by the safety governance systems implemented by the responsible clinical managers.

We were somewhat surprised by the participants’ honesty and willingness to discuss positive and negative aspects related to their own work in both part A and part B. The hazid approach thus may be defusing the fear often attributed to situations where own working processes are scrutinized.

The findings from part A and part B appear to be quite consistent. We think therefore that the validity is sufficiently high to report our experiences outside the organizations studied. The experiences reported from part B are also reproduced after introducing hazid as a routine in the system. Thus, we think that the findings from our real-life, case study approach can be claimed to be reliable, at least when the method is customized to the needs in the organization concerned.

The hazid seems to be a flexible tool for establishing real-time risk pictures with a sound internal validity for the systems investigated. The use of reliable methods for making and updating risk pictures for managerial purposes is not least important in times when risk factors are continuously changing.^19^

### Limitations

As with all case-based studies, it is a great challenge to assess the external validity of the findings. In this study, we have tried to compensate somewhat for this limitation by choosing two quite different supporting systems in two separate hospitals with broad clinical services as well as teaching and research. Hazid is a well-tested method in other settings, and in itself it need not be further validated. We believe this study shows that with some fine-tuning, it is possible to use this method in health care systems. We have not tried it in clinical settings, which will be the next step for validating hazid as a possible standard method in the patient safety tool box.

In both the studied systems, there was an underlying climate of trust between the employees and the managers. We presume that if used in a high-conflict environment, the hazid method, characterized by a quite open and inviting style, will lose some of its potential as the participants may not feel free to present their own opinions in safety.

## Conclusion

We claim that a hazid process is a meeting between competent workers, to be regarded as experts in their field, reflecting and elaborating on their own risk picture. This method generates other types of information to interpretations of quantitative or qualitative data from reporting systems related to deviations and incidents.

Using the hazid approach, relevant and specific risk factors can be described almost in real time by those performing the services. Suggestions related to possible corrective actions can be rapidly evaluated and implemented. By use of a follow-up hazid, the outcome of corrective actions can also be evaluated rapidly. Planning for and implementation of corrective actions in connection with conventional methods for risk analyses, for example, preliminary hazard analysis, appear to require long-term efforts.

Hazid appears to be a sensible method for engaging personnel working in supporting systems of hospital care provision. It may help clarifying which procedures and elements in practical work are perceived as high risk. The method seems appropriate to enhance the climate of trust encouraging the employees to present and reflect on their concerns. It can be used for presenting a risk picture as seen from the ground level in the organization. It may also be used for monitoring changes in the effects of current procedures at the practical level when external challenges are changing rapidly.
